# Pain Management in the Intensive Care Unit: Do We Need Special Protocols?

**DOI:** 10.5812/aapm.4523

**Published:** 2012-04-01

**Authors:** Mohammad Reza Hajiesmaeili, Saeid Safari

**Affiliations:** 1 Department of Anesthesiology and Critical Care Medicine, Rasoul-Akram Medical Center, Tehran University of Medical Sciences (TUMS), Tehran, Iran; 2 Pain Research Center, Shahid Sadoughi University of Medical Science and Health Services, Yazd, Iran

**Keywords:** Pain, Analgesia, Intensive Care Unit

Pain is a major public health issue throughout the world and represents a major clinical, social, and economic problem (1). The inability of intensive-care unit (ICU) patients to report pain because of mechanical ventilation, concomitant use of sedatives, or as a consequence of loss of consciousness should not preclude pain control. Acute pain has emerged as a leading stressor for ICU patients. Moderate to severe pain intensity has been observed in nearly 50% of ICU patients (2).

Various pharmacologic factors may increase the probability of excessive/prolonged effects in ICU patients including altered pharmacokinetic and pharmacodynamic characteristics with prolonged administration, altered protein binding, altered volume status, and end-organ dysfunction (3).

Special complications of inappropriate pain management in ICU are as follows: under-treatment leading to prolonged mechanical ventilation, increased ICU stay, hypoxemia, thromboembolic and pulmonary complications, self-removal of tubes and catheters, violence toward caregivers, patient-ventilator asynchrony, pain-related immune suppression, and readmission for further pain management, agitation, myocardial ischemia, delirium, and chronic pain. The complications of over-treatment for pain include prolonged mechanical ventilation and associated problems such as ventilator-associated pneumonia, post-traumatic stress disorder, prolonged cognitive impairment, unnecessary testing for altered mental status, prolonged ICU stay, skin breakdown, nerve compression, delirium, respiratory depression, brain or other neurologic injury, sedation, circulatory depression, urinary retention, impairment of bowel function, and sleep disruption (4-8).

It is strongly recommended that in patients with impaired cognition or concurrent diseases and in critically ill patients, additional interventions are needed for optimal pain management. A therapeutic trial of an analgesic should be considered in patients with increased blood pressure and heart rate or agitated behavior (4). 

The inclusion of pain management in the ICU checklist, as a part of daily rounds, can be a valuable tool for reducing the patient’s discomfort (9). Anesthesia-based pain services can improve the outcome and reduce the burden for ICU physicians and nurses (10). Thus, the optimal care of mechanically ventilated patients includes the use and integration of pain and sedation assessment tools to optimize the dosing of analgesic and sedative drugs.

Do patients undergoing different type of surgery have different need for analgesia?

A systematic pain assessment method for mechanically ventilated ICU patients could function as a criterion for good practice in the ICU (11).

The efficacy and outcomes of analgesic protocols for acute pain management have been studied extensively and are widely adopted. The latest practice guidelines were updated as the “Practice Guidelines for Acute Pain Management in the Perioperative Setting” in February 2012 and have been adopted by the ASA (4). These guidelines are published to facilitate the safety and effectiveness of acute pain management in the perioperative setting; decrease the risk of adverse outcomes; maintain functional abilities as well as physical and psychological well-being; and improve the quality of life (4). For acceptable guideline implementation, ongoing education and training are essential to preserve one’s skills, particularly when therapeutic approaches are modified (4).

Techniques for pain management include the following modalities:

1. Intermittent or continuous systemic opioids;
2. Multimodal techniques (administration of 2 or more drugs that act by different mechanisms to provide analgesia);
3. Central regional (i.e., neuroaxial) opioid analgesia; and
4. Peripheral regional analgesic techniques, including intercostal blocks, plexus blocks, and local anesthetic infiltration (4).

Techniques that reduce drug dosage (opioid-sparing effect) may be suitable for ICU patients. Behavioral modalities and techniques such as PCA that depend upon self-administration of analgesics are generally less suitable for the cognitively impaired.

Most patients on mechanical ventilation receive sedatives and analgesics without further evaluation (12). A prospective, multicenter, observational survey found that only 42% of patients underwent pain assessment on day 2 in the ICU, although 90% of patients were concomitantly given opioids. The survey concluded that pain assessment in patients on mechanical ventilation is independently associated with a decrease in hypnotic drug dosing, duration of mechanical ventilation, and duration of ICU stay (5, 11). Thus, pain assessment may be related to higher concomitant rates of sedation assessment and restricted use of hypnotic drugs.

Several scales and tools such as the visual analog scale, behavioral pain scale, and critical care pain observations are used for the assessment of analgesia and sedation in ICU. These scales and checklists for pain emphasize the complex nature of the problem.

The structured approaches and use of protocols to prevent the accumulation of drugs and metabolites that could lead to a slower recovery are supported by rapidly expanding evidence (7).

Awissi and coworkers concluded that establishing protocols for the management of sedation, analgesia, and delirium is a cost-effective practice and allows savings of nearly $1000 per hospitalization (13). Thus, we strongly recommend that strategies such as administering the right drugs in the right dose to the right patient at the right time for the right reasons should be defined to establish a practice protocol.
